# Impact of level five lockdown on the incidence of COVID-19: lessons learned from South Africa

**DOI:** 10.11604/pamj.2021.39.144.28201

**Published:** 2021-06-23

**Authors:** Felix Made, Wells Utembe, Kerry Wilson, Nisha Naicker, Nonhlanhla Tlotleng, Simbulele Mdleleni, Lusanda Mazibuko, Vusi Ntlebi, Phuti Ngwepe

**Affiliations:** 1 Epidemiology and Surveillance Section, National Institute for Occupational Health, National Health Laboratory Service, Johannesburg 2000, South Africa,; 2 Faculty of Health Sciences, School of Public Health, University of Witwatersrand, Johannesburg 2000, South Africa,; 3 Department of Toxicology, National Institute for Occupational Health, National Health Laboratory Service, Johannesburg 2000, South Africa,; 4 Department of Environmental Health, Faculty of Health Sciences, University of Johannesburg, Johannesburg 2000, South Africa,; 5 Foundation for Professional Development, Pretoria, South Africa

**Keywords:** Basic reproductive number, laboratory confirmed cases, estimated cases, forecast cases

## Abstract

**Introduction:**

the level five (L5) lockdown was a very stringent social distancing measure taken to reduce the spread of COVID-19 infections. This study assessed the impact of the L5 lockdown and its association with the incidence of COVID-19 cases in South Africa (SA).

**Methods:**

data was obtained from the National Department of Health (NDoH) from the 5^th^ March to the 30^th^ April 2020. A basic reproductive number (R0) and a serial interval were used to calculate estimated cases (EC). A double exponential smoothing model was used to forecast the number of cases during the L5 lockdown period. A Poisson regression model was fitted to describe the association between L5 lockdown status and incident cases.

**Results:**

a total of 5,737 laboratory-confirmed cases (LCC) were reported by 30^th^ April 2020, 4,785 (83%) occurred during L5 lockdown. Our model forecasted 30,629 cases of COVID-19 assuming L5 lockdown was not imposed. High incidence rates of COVID-19 were recorded in KwaZulu-Natal and Mpumalanga Provinces during the L5 lockdown compared to the other provinces. Nationally, the incident rate of COVID-19 was 68.00% higher in L5 lockdown than pre-lockdown for LCC.

**Conclusion:**

the L5 lockdown was very effective in reducing the incidence of COVID-19 cases. However, the incident rates of LCC and EC were higher nationally, and in some provinces during the L5 lockdown.

## Introduction

On 31^st^ December 2019, the Chinese public health authorities in the City of Wuhan reported cases of pneumonia of unknown aetiology [1]. The pneumonia was later linked to a virus called severe acute respiratory syndrome coronavirus 2 (SARS-CoV-2) [2]. A novel coronavirus was identified from sequencing lower respiratory tract samples of infected patients [1], and was subsequently named 2019 novel coronavirus (2019-nCoV) or COVID-19 virus [3]. Due to inadequate risk assessment and limited reporting, the virus spread rapidly across the world [4]. The World Health Organization (WHO) declared the outbreak a Public Health Emergency of International Concern and a pandemic on 30^th^ January and 11^th^ March 2020, respectively [5]. The WHO reported that as of 23^th^ August 2020, there was no specific treatment or vaccine for the virus [6], but various preventive measures were recommended, including washing hands with soap or using alcohol-based hand sanitizers regularly, using face masks in public spaces, and avoiding touching one´s face. Furthermore, the WHO recommended measures to “flatten the transmission curve” of COVID-19, including restricted travelling and social distancing by staying at home, also called 'lockdown' [6]. The aim of the lockdown was to suppress the community spread of SARS-CoV-2 and to disrupt the chain of transmission. However, despite the implementation of these preventive measures by many countries, the virus infected more than 3 090 445 people worldwide with an estimated 200 000 deaths by 30 April 2020 [7]. On the same day, Africa had 24,713 cases, of which 5,350 were from South Africa (SA) from 5^th^ March 2020, the date that the first case was confirmed [7].

The daily increase in the number of confirmed COVID-19 cases in SA led to the president declaring the outbreak as a National State of Disaster on 15^th^ March 2020 [8]. Shortly after, on 23^th^ March, a nationwide level five (L5) lockdown was announced, which commenced on 27^th^ March 2020 [9]. The L5 lockdown was a stringent measure to reduce the spread of the virus and included the closure of schools and businesses, restrictions on international and inter-provincial travel, and instructions to stay home. The only businesses that were allowed to operate were “essential services” which included healthcare services, food and grocery stores and pharmacies. The aim of the lockdown was for the government to have adequate time to prepare public healthcare facilities for the anticipated large number of cases. However, the number of cases continued to increase, although at a slower rate. On 9^th^ April 2020, the government reported that the average daily increase in the number of new cases had decreased from 42% in pre-lockdown to 4% during the L5 lockdown [10]. Apart from the decrease in the number of new cases, on the negative side, the consequences of the lockdown included loss of employment and suppression of freedom [11]. The long-term economic implications led to the SA government easing lockdown regulations in May 2020, to save the country´s economy [12]. However, evidence has shown that control measures, such as lockdowns, decreased the basic reproductive number (R0) - the average number of new cases generated by one infected case in a susceptible population - over time in China [13]. The R0 determines the extent of transmission in the presence and absence of control measures, and the ability of these measures to decrease spread [13].

**Objectives:** stringent social distancing measures like the L5 lockdown have been implemented by many countries. A previous study found that lockdown was effective in decreasing the number of new infections from COVID-19 among countries that implemented the measure compared to those that did not [14]. The trends conducted in 27 countries (both developed and developing) confirmed that the prevalence and mortality from COVID-19 tend to decline during lockdown [15]. Countries similar to South Africa which include India in terms of its socioeconomic status have imposed stringent lockdowns very early at the beginning of the pandemic, this has resulted in a significant reduction in the rates of transmission of COVID-19 infection [16]. Therefore, the objectives of this study were to assess the impact of the L5 lockdown in reducing the spread of COVID-19 from 27^th^ March to 30^th^ April 2020 and investigate its association with incident cases.

## Methods

**Study design, settings, and data sources:** this is a cross-sectional study. We analysed publically-available data on LCC of COVID-19 from the National Department of Health (NDoH), from 5^th^ March to 30^th^ April 2020. Data for the pre-lockdown period from 5^th^ to 26^th^ March 2020 were used to compare the impact of L5 lockdown. We also obtained data from each province for the number of tests conducted per day, gross domestic product (GDP) income per capita, most recent mid-year population estimates (for 2019), and the start-dates of community screening [17].

**Statistical methods:** a median serial interval of 4.60 days (95% CI: 3.50-5.90), which accounted for right truncation and most certain pairs of cases, was considered [18]. The serial interval was used as a cut-off date to exclude cases that were likely to occur after the L5 lockdown. This study assumed R0 of 2.95 (95% CI: 2.83-3.33) calculated from 5 to 26 March 2020, using SA COVID-19 data [19]. This R0 was used to estimate the number of new cases that occurred in pre-lockdown. Assuming that person-to-person contact was significantly reduced during the lockdown, we assumed a lower R0 of 1.40 provided by the WHO for human-to-human transmission to estimate the number of new cases that might have occurred during the lockdown [20]. The proportion of cases in each province was calculated, using the total number of cases in the country as a denominator. The average number of new cases per day in the first seven days (rolling moving average) was calculated over the L5 lockdown period. To assess the impact of the L5 lockdown in reducing the number of new cases of COVID-19, we forecasted or predicted the number of cases that would occur from 27^th^ March to 30^th^ April, the period of the lockdown. The forecast was conducted on the assumption that if L5 lockdown was not implemented, the number of cases would possibly increase exponentially. We used available data from the daily number of LCCs that were recorded from the 5^th^ to the 26^th^ March 2020, the period of the first confirmed case in South Africa, and before the L5 lockdown, to project cases that would possibly occur from the 27^th^ March to the 30^th^ April 2020, the period of the lockdown.

To forecast the number of cases, we adopted space-time series forecasting approaches using Holt’s trend double exponential smoothing from the space-time models to produce forecasts [21]. This model is the most accurate for forecasting the number of new COVID-19 infection when compared with other models [22]. Exponential smoothing models can capture trend and seasonality which can be additive or multiplicative to show forecasting patterns. In this study, we fitted the double exponential smoothing with the trend without seasonality. We assumed an exponential increase in the number of new COVID-19 cases and no seasonal variation given the short period of the L5 lockdown. Mathematically, the double exponential smoothing was done using the following equations.

$$\begin{array}{ll} Yt+1 & =Lt+\left(h\right)Tt\\ Lt & =\alpha Yt+\left(1-\alpha \right)\left(Lt-1+Tt-1\right)\\ Tt & =\beta \left(Lt-Lt-1\right)+\left(1-\beta \right)Tt-1 \end{array}$$

Where in equation 1, Yt+1 is the forecast for period t+1, in equation 2, Lt is the level value at time t, and in equation 3, Tt is the trend value at time t, α and β are the smoothing parameters, and h is the forecast horizon. We chose values of α and β as 0.9 and 0.8, respectively, for parameterization of the model. These values are close to 1, the value used to forecast COVID-19 [22]. Automatic selection of the most accurate forecasting models exists such as the use of information criteria. The information criteria are based on an optimized likelihood function with penalization for model complexity. In this study, we used a judgemental or discretionary model selection approach [23], for easy communication of the results with stakeholders, and to fully understand the COVID-19 data in South Africa. A Poisson regression model was also fitted to investigate the association between lockdown status and incident cases while adjusting for measures such as community screening, number of tests per day, GDP income per capita, mid-year population estimates, and province. Correlation between cases within the same province (cluster) was taken into account to calculate an unbiased estimate. Statistical significance was considered if p ≤ 0.05 and estimates were presented as odds and incidence rate ratios. The analysis was done using Geographic Information System (GIS), STATA Version 16.1 (4905 Lakeway Drive, College Station, TX, USA) and Microsoft Excel Spreadsheet.

## Results

**Figure 1:**
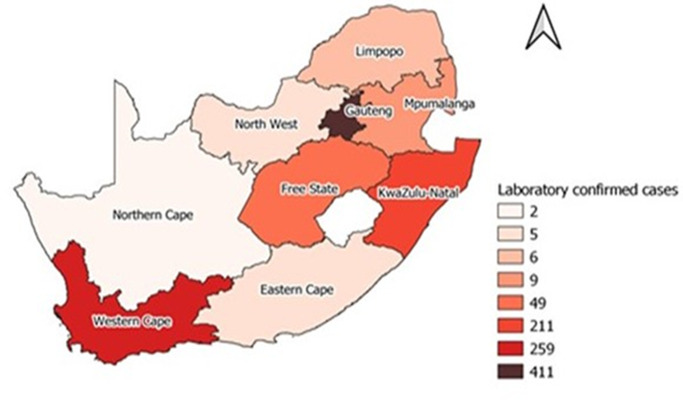
distribution of cases before L5 lockdown by provinces in SA

**Figure 2:**
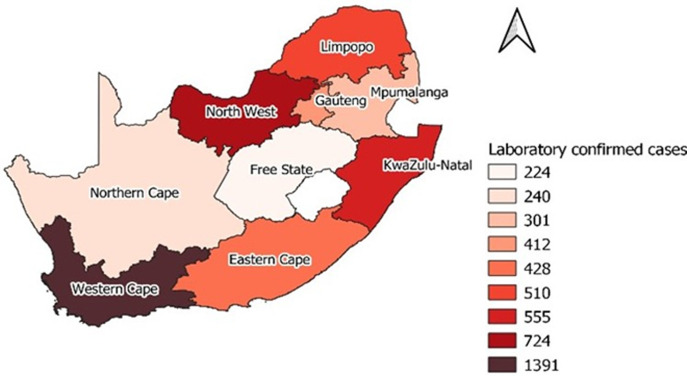
distribution of cases during L5 lockdown by province in SA

**Descriptive and outcome data:**Figure 1 and Figure 2 show the geographical distribution of confirmed cases by province, before and during the L5 lockdown in South Africa, respectively. A total of 5,737 cases was reported by 30^th^ April 2020, the last day of L5 lockdown, 4,785 (83%) occurred in L5 lockdown and 952 (17%) in pre-lockdown. Comparison of the number of cases in pre-lockdown and L5 lockdown generally showed an increase in each province. Gauteng Province recorded the highest number of cases (411), followed by the Western Cape Province (259) in pre-lockdown. During the L5 lockdown, the Western Cape Province, followed by KwaZulu-Natal Province, recorded the highest number of cases, 1,391 and 724, respectively. In both periods, the Northern Cape, Mpumalanga, and Limpopo Provinces recorded relatively lower numbers of cases.

**Figure 3:**
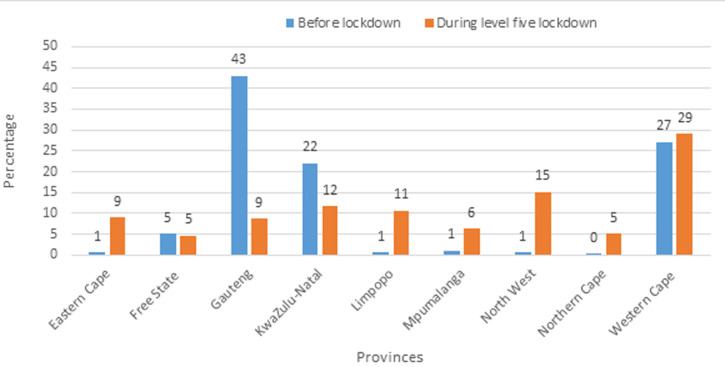
percentage distribution by province for laboratory-confirmed COVID-19 cases

**Figure 4:**
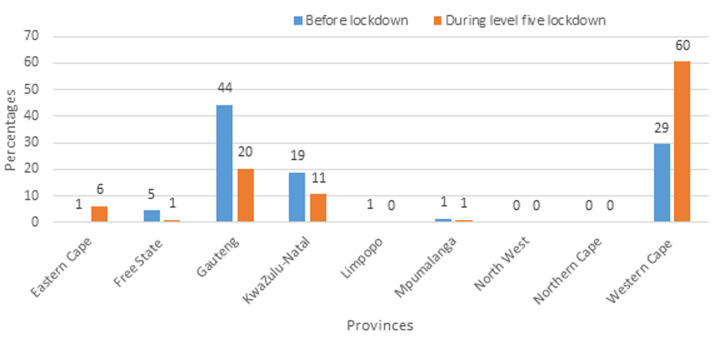
percentage distribution by province for estimated cases

**Figure 5:**
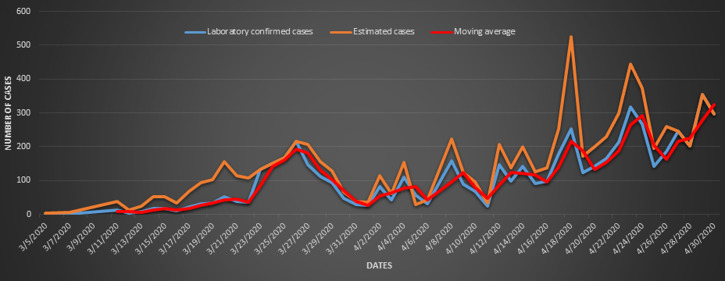
trends in the number of cases from the first day of infection to the end of L5 lockdown. Note: lockdown started on 27^th^ March 2020

**Main results:** the percentage distribution of COVID-19 cases by province for LCC and EC is shown in Figure 3 and Figure 4, respectively. Figure 3 shows that during L5 lockdown, Gauteng and KwaZulu-Natal Provinces had a high percentage decrease in the number of cases (from 43% to 9%, and from 22% to 12%, respectively). A slight increase in the proportion of cases was observed in the Western Cape Province during the lockdown, while other provinces, including the Eastern Cape (from 1% to 9%), Limpopo (from 1% to 11%), and the North West (from 1% to 15%) recorded high increases. Figure 4 displays the percentage distribution for EC, by province, in pre-lockdown and L5 lockdown. The Western Cape Province had a significantly higher percentage of EC, from 29.00% in pre-lockdown to 60.00% during L5 lockdown. The proportions of EC of COVID-19 decreased in Gauteng, KwaZulu-Natal and Free State Provinces during L5 lockdown. The distribution of cases in the remaining provinces, pre-and during the lockdown, were similar. Figure 5 shows trends in COVID-19 cases per day, for the entire country. There was a gradual increase in the number of daily LCCs from 21^st^ to 26^th^ March, before a rapid decline in the first week of L5 lockdown. After the first week of the lockdown, there was a fluctuation in the number of new COVID-19 cases observed until 30^th^ April. Similar trends were seen for the number of EC and the rolling moving average.

**Figure 6:**
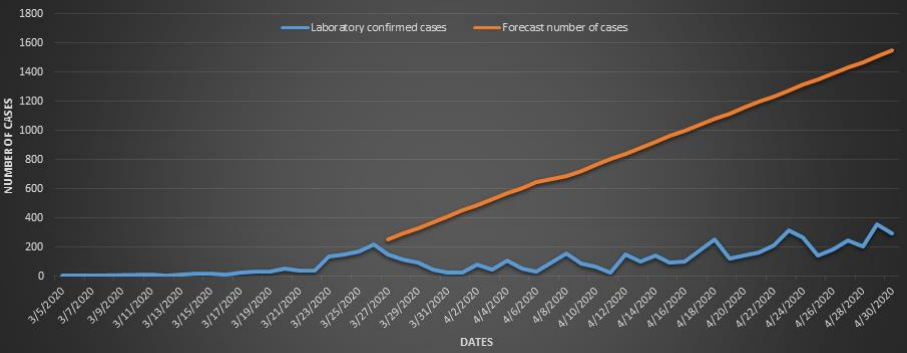
laboratory confirmed forecast numbers of cases of COVID-19 for the 34 days, the period of the L5 lockdown. Mean of LCC: 140 (95% CI: 110-171); mean of forecast number of cases: 901 (95% CI: 765-1037)

The COVID-19 LCC that occurred in the L5 lockdown from 27^th^ March to 30^th^ April were compared with the forecast number of cases for the same period as presented in Figure 6. From 5^th^ to 26^th^ March, we had recorded 18 days´ data points, while 4 days´ data points were missing or not reported. These data points were used to predict the number of cases that would occur in the period of the lockdown assuming no lockdown was initiated. The mean of LCC in the lockdown period was 140 (95% CI: 110-171), while for the forecast number of cases was 901 (95% CI: 765-1037). The forecast number of new COVID-19 cases for the period was 30 629, while the number of LCC during the lockdown was 4 785. This indicates a decrease of 25 844. The association between L5 lockdown and incidence of COVID-19 is shown in Table 1, nationally and by province. For LCC, there were statistically significant associations between L5 lockdown and the incidence of COVID-19 in KwaZulu-Natal and Mpumalanga Provinces. The KwaZulu-Natal Province had the highest incidence rate of COVID-19, which was three times high (95% CI: 1.28-7.42) during the L5 lockdown than in pre-lockdown for LCC, followed by Mpumalanga Province (IRR: 2.07, 95% CI: 1.15-3.74). The Eastern Cape and Free State Provinces had 80% higher rate of COVID-19 during L5 lockdown than in pre-lockdown with marginal significant differences. Apart from the Northern Cape, other provinces had a higher rate of COVID-19 cases during the L5 lockdown than in pre-lockdown but with differences that were not statistically significant. Nationally, the rate of COVID-19 was 68% (IRR: 1.68, 95% CI: 1.19-2.34) higher during L5 lockdown than in pre-lockdown for LCC. The incidence rates of COVID-19 cases in the Eastern Cape, KwaZulu-Natal, Limpopo and North West Provinces were statistically significantly higher during the L5 lockdown than in pre-lockdown. Nationally, the rate of COVID-19 was 38% (IRR: 1.38, 95% CI: 1.14-1.68) higher during L5 lockdown than in pre-lockdown.

**Table 1:** association between lockdown and incidence of COVID-19 (Poisson regression analysis)

SA province	LCC	EC
	IRR (95% CI)	IRR (95% CI)
	(Ref: pre-lockdown)	
National	1.68 (1.19-2.37)	1.38 (1.14-1.68)
Eastern Cape	1.93 (0.98-3.81)	1.53 (1.21-1.92)
Free State	1.86 (0.97-3.55)	1.46 (0.74-2.87)
Gauteng	1.39 (0.62-3.10)	1.19 (0.62-2.26)
KwaZulu-Natal	3.08 (1.28-7.42)	2.79 (1.20-6.45)
Limpopo	1.47 (0.78-2.78)	1.84 (1.22-2.78)
Mpumalanga	2.07 (1.15-3.74)	1.43 (0.78-2.64)
North West	1.10 (0.84-1.43)	1.77 (1.21-2.60)
Northern Cape	0.94 (0.64-1.38)	1.28 (0.79-2.05)
Western Cape	2.71 (0.81-9.03)	1.67 (0.80-3.45)

IRR: incidence rate ratio; CI: confidence interval; provincial estimate adjusted for the number of tests per day and community screening; National estimate adjusted for the provinces, number of COVID-19 tests done, community screening, provincial mid-year population estimates, and GDP-income per capita

## Discussion

The COVID-19 pandemic has caused unprecedented health and economic challenges around the world. As of 12^th^ August 2020, more than 20 million people were infected worldwide, while 737,417 had died; 566,109 cases were from South Africa [24]. To reduce the spread of COVID-19 infections, countries have implemented lockdown regulations which include restriction of movement, travelling, and closing schools and businesses. These restrictions were also implemented by South Africa in five different levels, according to the strictness in social distancing. It is important to note that the aim of a lockdown is not to prevent or stop COVID-19 infections but to slow down person-to-person transmission. In this study, we assessed the impact of L5 lockdown and its association with the incidence of COVID-19, by comparing the number of cases, forecasting, and incidence rate ratios within provinces in SA before lockdown and during the L5 lockdown.

**Key outcomes:** we adopted the R0 of 2.95 derived from cases that occurred in pre-lockdown in South Africa and assumed a much lower R0 of 1.4 during the lockdown since it is expected that lockdown reduces contact rates, thus also reducing the rate of transmission. The reported values range from 1.4 to 6.49, with an average of 3.00 [25]. However, since the lockdown and other restriction strategies are aimed at decreasing the contact rate, a change in the R0 value is expected. The value of 1.4 used in this study is much higher than the one observed in France where a value of 0.47 (95% CI: 0.45-0.50) was calculated during lockdown compared to the estimates calculated at the early stage of the epidemic [26]. Most importantly, the R0 of 1.4 is above the threshold value of 1.00 which implies that the number of cases stays the same. Our results show that there were 4 785 new cases (an increase of 83%) during L5 lockdown, compared to pre-lockdown (952). Identification of cases through contact tracing improved during the L5 lockdown which might have helped in maintaining the number of new cases at a fairly constant level. Towards the end of the lockdown, the number of new cases increased, as the government implemented community screening, identification of symptomatic individuals, and testing. The Gauteng and the Western Cape Provinces had the highest number of cases in pre-lockdown. During the L5 lockdown, both the Western Cape and KwaZulu-Natal Provinces had the most number of cases. In the provinces with a low number of cases in L5, transmission rates might have fallen because of possible reductions in contact rates and improved hospital infection controls measures, and an increase in screening and hospital attendance by symptomatic individuals. For LCC, the percentage increase in the number of cases during the L5 lockdown could be linked to the increased number of tests conducted per day in the Western Cape Province, and contravention of lockdown rules by superspreaders. Superspreading events can be driven by many factors, including poor hygiene practice, social customs that contradict the regulations, poor health-seeking behaviour, and non-adherence to public health measures [27].

For the EC, L5 lockdown had a positive impact in reducing the incidence of COVID-19 in six of the nine provinces. The restricted movement within and between provinces might have reduced the number of incident cases. However, 80% of the EC during L5 lockdown were in the Western Cape and Gauteng Provinces, compared to 38% of the LCC. The incubation period which varies between 5 and 12 days from the day of infection may affect the EC [28]. There appears to be variation in the number of new cases that are often characterised by so-called superspreading events [29]. In this regard, R0, as a mean or median value, might have failed to capture the heterogeneity of transmission among infected persons over time [30]. Superspreaders can comprise approximately 10% of cases that are responsible for up to 80% of transmission [31]. This value is consistent with our results which showed an increase in infections in some provinces. The trend in the number of new cases showed a gradual decrease in the first week of the L5 lockdown, with some fluctuations from the third week. The gradual decrease in the number of new cases could be due to compliance with L5 lockdown regulations. Another South African study also found an immediate decrease in the number of new cases at the start of the lockdown [32]. More notably, testing coverage decreased just after the lockdown [32], which could partially explain the reduction in new cases.

**Interpretation:** our findings are similar to those from a study in Wuhan, China, where local transmission was reduced immediately after extensive control measures were enforced, akin to the L5 lockdown [33,34]. However, our findings are not consistent with those from Italy and the United States, where there were peaks a few weeks after lockdown; followed by a decrease in the sustained lockdown [35,36]. Also, in Guangdong Province in China, the reduction in the incidence of COVID-19 started one week into the lockdown [37], unlike in our study. The increase in the numbers of new cases immediately after a lockdown in these countries could have been brought about by unknown cases that increase contact rates within households [38], which may not be the case in South Africa due to the country´s early response. Furthermore, the number of tests conducted was probably not constantly increasing per day in South Africa.

In this study, we forecasted the number of COVID-19 cases that would occur during the period of L5 lockdown, assuming no lockdown. We then compared the forecast with the actual number of LCC during the lockdown. Our study found that there was a decrease of 25 844 cases during L5 lockdown where only 4 785 LCC were reported, indicating that the lockdown was effective in reducing the number of new cases of COVID-19. A cross-country comparative study also reported that lockdown was effective in reducing the number of new cases [14]. The high predicted number of new infections assuming L5 was not implemented may also be supported by the recent dramatic rise in the number of LCC after the L5 lockdown was lifted by the South Africa government [39]. A forecast number of cases may help in decision making about the implementation of lockdown levels. Our finding suggests that the number of new COVID-19 cases would have continued to increase exponentially if there was no L5 lockdown that involved mandatory stringent social distancing measures. This is consistent with other countries such as Iran, Spain, France, Italy and the United Kingdom, where a 25% increase in cases was expected without imposing the lockdown [22].

We also investigated the association between the number of COVID-19 cases and the L5 lockdown, after adjusting for possible confounding factors. We calculated higher incidence rates of COVID-19 in KwaZulu-Natal and Mpumalanga Provinces during the L5 lockdown than before lockdown, for LCC. For the EC, the KwaZulu-Natal Eastern Cape, Limpopo and the North West Provinces had higher incidence rates during the lockdown than in pre-lockdown. Nationally, the incidence rate of COVID-19 was higher during L5 lockdown than in pre-lockdown. The high incidence rates in some of these provinces, and nationally, might have been caused by the asymptomatic members of the population that were infected just before the L5 lockdown and tested after several days into the lockdown after becoming symptomatic. A previous study found that the percentage of symptomatic patients was very high, at 51.4% to 69% during the lockdown [40]. The introduction of community screening, increased contact tracing, and awareness of COVID-19 [41], were likely not implemented at the same time across provinces, could have contributed to the increased incidence rates observed during the L5 lockdown. The L5 lockdown aimed to reduce the R0 to a level that would substantially slow down the rate of increase in incident cases. A previous study has confirmed that such stringent measures reduced the R0 to below 1. [42]. However, our study did not adopt a R0 < 1.00, as this would mean infections die out which was not observed in the L5 lockdown. The WHO reported that almost 80% of transmission takes place in household settings [43]. This might explain the high incidence rates observed nationally and in some provinces.

**Limitations:** a limitation of this study is the risk of poor quality of data from inaccurate reporting. Also, we used a high R0 in our analysis, which could have varied greatly over the period of the study.

**Funding:** this research did not receive any specific grant from funding agencies in the public, commercial, or not-for-profit sectors.

## Conclusion

The number of new COVID-19 cases kept increasing during the L5 lockdown compared to the period before the implementation of the lockdown. The forecast number of new cases indicated that L5 lockdown reduced the number of new infections significantly. However, the incident rate ratios of LCC and EC were higher nationally, and in some provinces such as the Western Cape and Eastern Cape. This study has highlighted that L5 lockdown was very effective in reducing the number of new infections. Therefore, future decisions or policies to control the spread of a new epidemic should include measures such as those included in the L5 lockdown.

### What is known about this topic

- The L5 lockdown consisted of stringent social distancing measures put in place to reduce the rate of COVID-19 infection;
- Lockdown regulations disrupt the chain of transmission, thus reduce the rate of COVID-19 infection.

### What this study adds

- This study emphasised the importance of L5 lockdown as a significant reduction in the number of new COVID-19 cases was observed;
- Adherence to lockdown rules is not always followed by some individuals, this creates superspreading events.
